# Pharmacokinetic Interactions of Herbs with Cytochrome P450 and P-Glycoprotein

**DOI:** 10.1155/2015/736431

**Published:** 2015-01-06

**Authors:** Hyun-Jong Cho, In-Soo Yoon

**Affiliations:** ^1^College of Pharmacy, Kangwon National University, Chuncheon 200-701, Republic of Korea; ^2^College of Pharmacy and Natural Medicine Research Institute, Mokpo National University, 1666 Youngsan-ro, Muan-gun, Jeonnam 534-729, Republic of Korea

## Abstract

The concurrent use of drugs and herbal products is becoming increasingly prevalent over the last decade. Several herbal products have been known to modulate cytochrome P450 (CYP) enzymes and P-glycoprotein (P-gp) which are recognized as representative drug metabolizing enzymes and drug transporter, respectively. Thus, a summary of knowledge on the modulation of CYP and P-gp by commonly used herbs can provide robust fundamentals for optimizing CYP and/or P-gp substrate drug-based therapy. Herein, we review ten popular medicinal and/or dietary herbs as perpetrators of CYP- and P-gp-mediated pharmacokinetic herb-drug interactions. The main focus is placed on previous works on the ability of herbal extracts and their phytochemicals to modulate the expression and function of CYP and P-gp in several *in vitro* and *in vivo* animal and human systems.

## 1. Introduction

In the last decade, a number of herbal products have attracted growing interest as a complementary and alternative medicine for the prevention and treatment of various diseases [1]. Recent surveys have reported that the prevalence of herbal medicine use is approximately 20% and the concurrent use of herbal medicine occurs in 20–30% of prescription drug users in the United States [2–4]. Herbal products have been generally considered as natural and safe. However, some of their constituents can modulate various xenobiotic metabolism and transport systems which play a significant role in the absorption and disposition of prescription drugs. Therefore, drug metabolizing enzymes and drug transporters-mediated herb-drug interactions can occur frequently in drug- and/or herb-based therapies [1, 5].

Phase I metabolism generally results in the introduction of a hydrophilic functional group into molecules or the unveiling of new functional groups of molecules. It includes various reaction types such as oxidation, reduction, and hydrolysis. Cytochrome P450 (CYP) monooxygenase is a superfamily of hemoproteins responsible for the phase I metabolism of various xenobiotics and some endogenous substances such as steroids [6]. Although CYP is ubiquitously expressed in a number of organs, most of drug metabolizing CYP isoforms are expressed at the highest level in the liver [7]. Approximately 70–80% of all currently prescribed drugs are metabolized by the CYP system [8]. P-glycoprotein (P-gp), also known as multidrug resistance protein, is an ATP-dependent efflux pump with broad substrate specificity [9, 10]. P-gp is highly expressed in the apical (luminal) membrane of intestinal epithelium, hepatocytes, kidney proximal tubule epithelium, and brain capillary endothelium, where it pumps a variety of xenobiotics into the intestinal lumen, bile duct, renal tubule, and brain capillary, respectively [11, 12]. It plays an important role in the intestinal absorption, distribution to the central nervous system, and biliary/urinary excretion of drugs [13]. Therefore, the inhibition or induction of CYP and/or P-gp by concurrent herbs may result in pharmacokinetic interactions potentially leading to therapeutic failure [14]. On the other hand, the herbal modulation of their expression and/or activity could be a useful strategy to improve the efficacy and safety of CYP and/or P-gp substrate drugs [14].

Here, this paper reviews some of the commonly used medicinal and/or dietary herbs as perpetrators of CYP- and P-gp-mediated pharmacokinetic drug interactions. The main focus is placed on a current understanding on the effect of selected herbs and their phytochemicals on the expression and activity of CYP and P-gp in several* in vitro* and* in vivo* animal and human systems. St. John's Wort and grapefruit are not addressed in this paper, because they are well-documented as CYP and P-gp modulators in many previous reviews.

## 2. Herbal Modulations of CYP and P-gp

### 2.1. *Ginkgo biloba*

**Figure 1:**
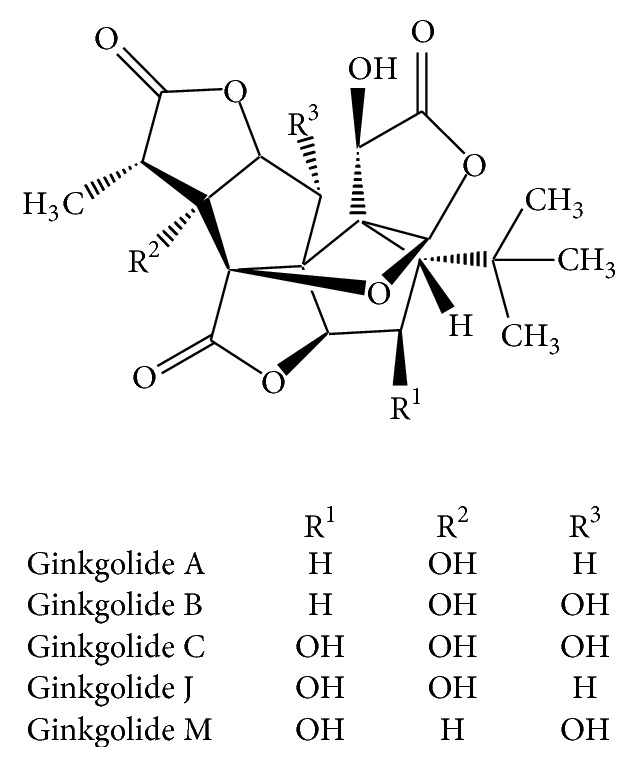
Chemical structures of ginkgolides.

*Ginkgo biloba* (ginkgo), also called as maidenhair tree, is 190 million years old and unique species of dioecious tree with no closing living relatives [1, 15]. The extracts of the ginkgo leaves have been widely used as a phytomedicine in Europe and as a dietary supplement in the United States [6]. The main pharmacologically active phytochemicals of the ginkgo extracts include flavonoid glycosides (e.g., quercetin, kaempferol, and isorhamnetin) and unique terpene lactones (ginkgolides; Figure 1) [6]. Ginkgolides are potent inhibitors of platelet activating factor, and ginkgo extracts are used for the treatment of cerebrovascular dysfunctions, dementia, memory impairment, and peripheral vascular disorders [16, 17]. The recommended dose of ginkgo is 120–240 mg/day for the treatment of dementia and memory impairment [1].

The effect of ginkgo and its main active components on the expression and activity of CYP and P-gp is listed in Table 1. In rats, the mRNA levels of CYP2B1/2 and 3A1/2 were significantly increased in rats treated with ginkgo extract during 4 weeks, while those of CYP1A1/2, 2C11, 2E1, and 4A1 were not significantly changed [18]. However, in another study, the* in vivo* activity of CYP1A2 was significantly increased in rats, which is not consistent with the mRNA results [19]. In human, the* in vivo* activity of CYP3A4 was increased, while the* in vitro* activities of CYP1A2, 2C9, and 2E1 were decreased by ginkgo extract [20, 21]. The* in vitro* or* in vivo* activity of human P-gp was significantly reduced by ginkgo extract [22, 23]. The activity of CYP1A2 and 2C9 was not significantly changed by ginkgolides, while that of CYP3A4 was increased by ginkgolide A* via* pregnane X receptor [24, 25].

### 2.2. *Allium sativum* and* Allium cepa*

**Figure 2:**
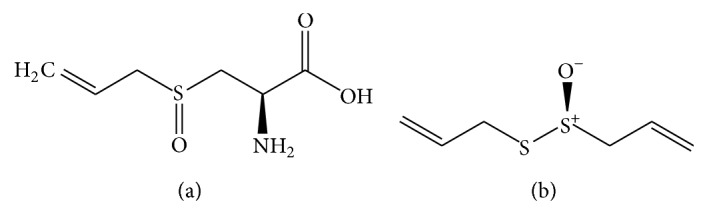
Chemical structures of alliin (a) and allicin (b).

*Allium sativum* (garlic) is a widely used medicinal and dietary herb which has antioxidant, antibacterial, hepatoprotective, hypolipidemic, antihypertensive, antiplatelet, procirculatory, antidiabetic, anticancer, and immunoenhancing efficacy [1, 26]. Garlic bulbs and cloves are mainly used for pharmaceutical products in the form of powder, oily preparation, or aqueous alcoholic extract [1, 27]. The main pharmacologically active phytochemicals of garlic include alliin (Figure 2(a)) and allicin (Figure 2(b)), diallyl disulphide, and diallyl sulphide [28]. Alliin is metabolized to allicin, and it is subsequently degraded to various organosulfur compounds including diallyl disulphide and diallyl sulphide which are believed to be mainly responsible for the beneficial biological effects of garlic [29]. The recommended dose of fresh garlic is about 4 g/day which is equivalent to about 8 mg garlic oil or 600–900 mg garlic powder daily standardized to 1.3% alliin content [1].

The effect of garlic and its main active components on the expression and activity of CYP and P-gp is listed in Table 2. In mouse, the administration of garlic juice for 8 days induced the protein expression of CYP1A2 and 2E1 [30]. Garlic extract inhibited* in vitro *CYP2C9^*^1, 2C19, 3A4, 3A5, and 3A7 activity, while it did not affect the CYP2D6 activity, and increased CYP2C9^*^2 activity in recombinant human CYP isozyme system [31]. A few divergent results on the modulation of P-gp by garlic have been reported as follows: a previous study reported the inhibitory effect of garlic extract on P-gp activity [31]; another study suggested the inductive effect of garlic extract on intestinal P-gp activity [32, 33]; P-gp activity in human CD4 cells was not affected by garlic extract without allicin [34]. Diallyl disulfide induced* in vivo* CYP2B1/2 activity in rats and inhibited* in vitro* CYP2E1 activity in recombinant rat and human CYP isozyme system [35, 36]. Allicin also inhibited* in vitro* CYP1A2 activity in recombinant human CYP isozyme system [36].

*Allium cepa* (onion) is a daily diet and has been used as a medicinal and dietary supplement for the treatment of hypertension and hyperlipidemia [37]. Onion extract possesses several pharmacological activities including antihypertensive, hypolipidemic, antithrombotic, antioxidant, antibacterial, and anticancer effects [38, 39]. The main pharmacological component of onion is known to be quercetin [37]. Onion extract did not significantly change P-gp-mediated efflux of rhodamine-123 in the everted rat gut sac system [37]. However, quercetin inhibited P-gp-mediated efflux of ritonavir in Caco-2 cells and human CYP3A4 activity in the Vivid assay kit system, while prolonged exposure of quercetin increased the mRNA expression of both P-gp and CYP3A4 in Caco-2 cells [40].

### 2.3. *Camellia sinensis*

*Camellia sinensis* (green tea) is used worldwide as a medicinal and dietary herb. Its leaves are consumed as a beverage, and its purified extract has been approved as a botanical drug by United States Food and Drug Administration (US FDA) [33]. A typical green tea beverage contains 30–42% dry-weight catechins which are the main pharmacologically active phytochemicals of green tea [47]. Epigallocatechin gallate (EGCG) is known as the most abundant catechin of green tea [10]. Numerous studies reported that green tea has anticancer, anti-inflammatory, chemopreventive, antimetastatic, and vasculoprotective properties [48–50]. The effect of green tea and its main active components on the expression and activity of CYP and P-gp is listed in Table 3. In rats, the administration of green tea extract increased the* in vivo* activity of CYP1A, 2B, and 3A [41–43]. In human, green tea extract inhibited CYP2C9, 2D6, and 3A4 activities in human liver microsomes [43], while it induced the mRNA and protein expression of CYP1A2 in LS-180 cells and CYP1A1/2 in Caco-2 cells [44]. Moreover, EGCG inhibited CYP1A2 and 3A4 activity in the same cell lines [44]. Green tea polyphenols including EGCG also inhibited P-gp-mediated efflux activity in multidrug-resistant CH(R)C5 [45] and KB-A1 cells [46].

### 2.4. *Glycyrrhiza glabra*

**Figure 3:**
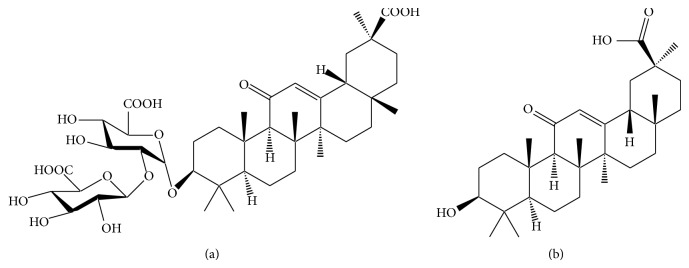
Chemical structures of glycyrrhizin (a) and glycyrrhetic acid (b).

The root of* Glycyrrhiza glabra* (licorice) is used as a herbal medicine for the treatment of peptic ulcer and cough and as a food additive for sweetening candies, beverages, and chewing gums [6, 51]. The main phytochemicals of licorice include glycyrrhizin (Figure 3(a)), liquiritigenin, coumarins, stilbenoids, fatty acids, phenols, and sterols [52]. Glycyrrhizin is metabolized by intestinal flora into its pharmacologically active form, glycyrrhetinic acid (Figure 3(b)). Licorice is known to have antimalarial, ulcer-healing, immunosuppressive, antihepatotoxic, antianemic, and anti-inflammatory properties [6, 53]. In mice and rats, multiple oral doses of licorice extract during 4 or 10 days induced the mRNA and protein expression of CYP3A and activity of CYP1A2, 2B1, and 3A [54]. Concurrent administration of glycyrrhizin altered the oral pharmacokinetics of midazolam in healthy male subjects possibly by a modest induction of CYP3A4 [55]. Moreover, glycyrrhetinic acid inhibited P-gp-mediated efflux of daunorubicin in P-gp-overexpressing KB-C2 cells [56].

### 2.5. *Zingiber officinale*

**Figure 4:**
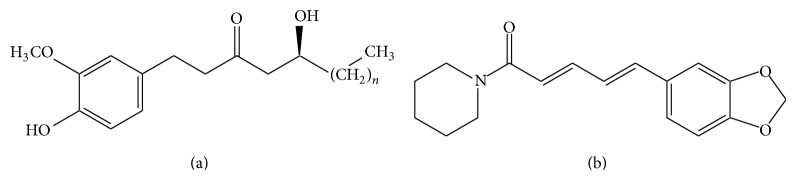
Chemical structures of gingerols (a) and piperine (b).

*Zingiber officinale* (ginger) has been widely used for the treatment of nausea and dyspepsia [57]. It acts as an agonist of cholinergic receptors expressed in the gastrointestinal tract, which is believed to be a mechanism of its prokinetic effect [58]. Moreover, ginger has several pharmacologic activities such as antiplatelets, antioxidant, antitumor, antivirus, antihepatotoxicity, and anti-inflammation [59]. The main pharmacologic component of ginger is gingerols (Figure 4(a)) which have a pungent flavor [60]. Ginger extract inhibited CYP2C9 and 3A4 activities in recombinant human CYP isozyme system [61] and CYP2C19 activity in human liver microsomes [62]. 6-gingerol inhibited P-gp-mediated efflux of daunorubicin and rhodamine-123 in KB-C2 cells [63], while it did not significantly change the* in vitro* activity of human CYP1A2, 2C9, 2D6, and 3A4 [64].

### 2.6. *Piper nigrum*

*Piper nigrum* (pepper) is widely used as a food ingredient with spicy taste [28]. The main active component of pepper is alkaloid piperine (Figure 4(b)) which is also used as a dietary supplement [65]. Piperine has several beneficial properties including antidiarrhoeal, chemopreventive, anti-inflammatory, antioxidant, and immunoenhancing activities [66–70]. Moreover, piperine may act as a bioavailability enhancer by inhibiting gastric emptying and gastrointestinal transit [71]. Bioperine is a commercial herbal product containing a minimum of 98% pure alkaloid piperine extracted from the fruits of black pepper [72]. It enhanced the oral bioavailability of coenzyme Q10 after single or multiple (14 and 21 days) dosing in twelve healthy adult male subjects [73]. The protein expression of hepatic CYP1A and 2B increased, while that of CYP2E1 decreased following multiple intraperitoneal injections of piperine in rats [74]. Piperine inhibited CYP3A4-mediated metabolism of verapamil in human liver microsomes and P-gp-mediated efflux of digoxin and cyclosporine in Caco-2 cells [75].

### 2.7. *Rosmarinus officinalis*

**Figure 5:**
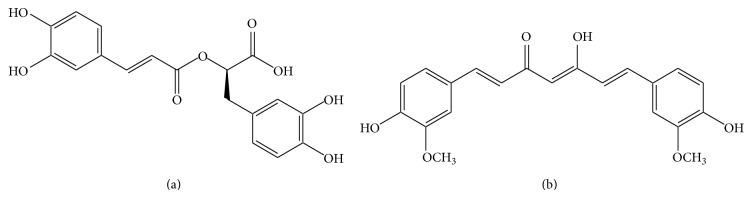
Chemical structures of rosmarinic acid (a) and curcumin (b).

*Rosmarinus officinalis* (rosemary) has been used as a dietary herb for beverage, flavouring food, and cosmetics and as a medicinal herb for stimulant, antirheumatic, diuretic, analgesic, antiepileptic, and cancer prevention [76]. The main active component of rosemary is rosmarinic acid (Figure 5(a)) [77]. The effect of rosemary and its main active component on the expression and activity of CYP and P-gp is listed in Table 4. Oral treatment with rosemary extract increased the protein expression of hepatic CYP2B1/2, while it did not change hepatic CYP1A1/2 in rats [78]. Moreover, rosemary extract inhibited P-gp-mediated efflux of doxorubicin and vinblastine in P-gp-overexpressing MCF-7 cells [79]. Rosmarinic acid induced the* in vitro* activity of CYP1A, 2B, and 3A in rat HepG2/C3A and MH1C1 cells [80]. However, it inhibited human recombinant CYP3A4 activity, but not CYP2C9 and 2D6 activities [81]. It reduced the mRNA and protein expression of P-gp and also inhibited P-gp-mediated efflux of doxorubicin and rhodamine 123 in SGC7901/Adr cells [82].

### 2.8. *Curcuma longa*

*Curcuma longa* (turmeric) has been widely used as food additives, cosmetics, and medical preparations for stomach upset, inflammation, skin wound, and tumor [10, 88]. The main active component of turmeric is curcumin (Figure 5(b)). Curcumin has antioxidant, anti-inflammatory, hypolipidemic, and anticancer activities, which may be attributed to its inhibitory effect on several cell signal transduction pathways [89–92]. In rats, the protein expression of intestinal CYP3A and P-gp was significantly reduced by treatment with curcumin at a dose of 60 mg/kg/day for 4 days [93]. Moreover, the protein expression of P-gp in primary cultured rat hepatocytes was reduced following 72 h cultures with curcumin [94]. Curcumin inhibited the activity of CYP1A1, 1A2, and 2B1 in rat liver microsomes [95]. In Caco-2 cells, curcumin reduced the mRNA expression and activity of P-gp, while turmeric extract increased these parameters [96]. However, both curcumin and turmeric extract reduced the protein expression and activity of CYP3A4 without affecting its mRNA expression in dihydroxyvitamin D3-treated Caco-2 cells [97].

### 2.9. *Panax ginseng*

*Panax ginseng* (ginseng) is widely used as a medicinal herb. It has various beneficial activities such as antihypertensive, antifatigue, antioxidative, hypolipidemic, immunoenhancing, and chemopreventive effects [6]. It is the 5th best-selling herb in the United States [98]. The major pharmacologically active components of ginseng are ginsenosides (triterpenoid dammarane saponins) [28]. There are approximately 12 different types of ginsenosides identified [33]. The recommended dose of ginseng is 200 mg/day of standardized extract containing 4% total ginsenosides [1]. The effect of ginseng and its main active components on the expression and activity of CYP and P-gp is listed in Table 5. In rats, ginseng extract inhibited the* in vitro* activity of CYP1A1/2, 1B1, and 2E1 in rat liver microsomes [83], while it did not affect the mRNA expression of rat hepatic CYP1A2, 2B1, and 3A23 [84]. Moreover, ginseng extract inhibited CYP1A1, 1A2, and 1B1 activities in recombinant human CYP isozyme system [85]. Ginsenoside Rd weakly inhibited CYP2C9, 2C19, 2D6, and 3A4 activities, while ginsenosides Rc and Rf increased CYP2C9 and 3A4 activities in recombinant human CYP isozyme system [86]. Ginsenoside Rg3 inhibited P-gp-mediated efflux in multidrug-resistant human fibroblast carcinoma KBV20C cells possibly by decreasing membrane fluidity [87].

## 3. Conclusions

The modulations of CYP and P-gp by ten herbs and relevant phytochemicals have been comprehensively reviewed. Evidences from* in vitro* and* in vivo* studies have indicated that herbs can interact with CYP isoforms and P-gp as inhibitors and/or inducers. Since the herbal modulation of CYP and P-gp may have significant clinical and toxicological implications, rigorous evaluation for the possibility of herb-drug interactions may be required in the development process of herbal medicines. Efforts to facilitate communications among patients and clinicians regarding a clinical risk of herb-drug interactions are also encouraged [28, 99]. Continuous improvements in our understanding on herb-drug interactions and their pharmacokinetic mechanisms will enable us to better predict, evaluate, and manage potential risks associated with a concurrent use of herb and drug-based therapies.

## Figures and Tables

**Table 1:** The modulation of CYP and P-gp by ginkgo.

Compound type	Species	System	CYP	P-gp	Ref.
Extract	Rat	*In vivo* mRNA	CYP1A1/2 (↔) CYP2B1/2 (↑) CYP2C11 (↔) CYP2E1 (↔) CYP3A1/2 (↑) CYP4A1 (↔)		[18]

Extract	Rat	*In vivo* Activity	CYP1A2 (↑)		[19]

Extract	Human	*In vitro* Activity	CYP1A2 (↓) CYP2C9 (↓) CYP2E1 (↓)		[20]

Extract	Human	*In vivo* Activity	CYP3A4 (↑)		[21]

Extract	Human	*In vitro/in vivo* Activity		P-gp (↓)	[22, 23]

Ginkgolides	Human	*In vitro* Activity	CYP1A2 (↔) CYP2C9 (↔)		[24]

Ginkgolide A	Human	*In vitro* Activity	CYP3A4 (↑)		[25]

**Table 2:** The modulation of CYP and P-gp by garlic.

Compound type	Species	System	CYP	P-gp	Ref.
Extract	Mouse	*In vivo* Protein	CYP1A2 (↑) CYP2E1 (↑)		[30]

Extract	Human	*In vitro* Activity	CYP2C9^*^1 (↓) CYP2C9^*^2 (↑) CYP2C19 (↓) CYP2D6 (↔) CYP3A4 (↓) CYP3A5 (↓) CYP3A7 (↓)		[31]

Extract	Human	*In vitro* Activity		P-gp (↓)	[31]

Extract	Human	*In vivo* Activity		P-gp (↑)	[32, 33]

Extract w/o allicin	Human	*In vitro* Activity		P-gp (↔)	[34]

Diallyl disulfide	Rat	*In vitro/in vivo* Activity	CYP2B1/2 (↑) CYP2E1 (↓)		[35]

Diallyl disulfide	Human	*In vitro* Activity	CYP2E1 (↓)		[36]

Allicin	Human	*In vitro* Activity	CYP1A2 (↓)		[36]

**Table 3:** The modulation of CYP and P-gp by green tea.

Compound type	Species	System	CYP	P-gp	Ref.
Extract	Rat	*In vivo* Activity	CYP1A (↑) CYP2B (↑) CYP3A (↑)		[41–43]

Extract	Human	*In vitro* Activity	CYP2C9 (↓) CYP2D6 (↓) CYP3A4 (↓)		[43]

Extract	Human	*In vitro* mRNA/protein	CYP1A1 (↑) CYP1A2 (↑)		[44]

EGCG	Human	*In vitro* Activity	CYP1A2 (↓) CYP3A4 (↓)		[44]

Catechins	Human	*In vitro* Activity		P-gp (↓)	[45]

EGCG	Human	*In vitro* Activity		P-gp (↓)	[46]

**Table 4:** The modulation of CYP and P-gp by rosemary.

Compound type	Species	System	CYP	P-gp	Ref.
Extract	Rat	*In vivo* Protein	CYP1A1/2 (↔) CYP2B1/2 (↑)		[78]

Extract	Human	*In vitro* Activity		P-gp (↓)	[79]

Rosmarinic acid	Rat	*In vitro* Activity	CYP1A (↑) CYP2B (↑) CYP3A (↑)		[80]

Rosmarinic acid	Human	*In vitro* Activity	CYP2C9 (↔) CYP2D6 (↔) CYP3A4 (↓)		[81]

Rosmarinic acid	Human	*In vitro* mRNA/protein Activity		P-gp (↓)	[82]

**Table 5:** The modulation of CYP and P-gp by ginseng.

Compound type	Species	System	CYP	P-gp	Ref.
Extract	Rat	*In vitro* Activity	CYP1A1/2 (↓) CYP1B1 (↓) CYP2E1 (↓)		[83]

Extract	Rat	*In vitro* mRNA	CYP1A2 (↔) CYP2B1 (↔) CYP3A23 (↔)		[84]

Extract	Human	*In vitro* Activity	CYP1A1 (↓) CYP1A2 (↓) CYP1B1 (↓)		[85]

Ginsenoside Rd	Human	*In vitro* Activity	CYP2C9 (↓) CYP2C19 (↓) CYP2D6 (↓) CYP3A4 (↓)		[86]

Ginsenoside Rc, Rf	Human	*In vitro* Activity	CYP2C9 (↑) CYP3A4 (↑)		[86]

Ginsenoside Rg3	Human	*In vitro* Activity		P-gp (↓)	[87]
